# Visual Saliency Detection for Over-Temperature Regions in 3D Space via Dual-Source Images

**DOI:** 10.3390/s20123414

**Published:** 2020-06-17

**Authors:** Dawei Gong, Zhiheng He, Xiaolong Ye, Ziyun Fang

**Affiliations:** School of Mechanical and Electrical Engineering, University of Electronic Science and Technology of China, Chengdu 611731, China; pzhzhx@126.com (D.G.); yxl198868@foxmail.com (X.Y.); f459534668@126.com (Z.F.)

**Keywords:** component, robot work, object detection, adaptive sampling, surface mapping, coordinate mapping

## Abstract

To allow mobile robots to visually observe the temperature of equipment in complex industrial environments and work on temperature anomalies in time, it is necessary to accurately find the coordinates of temperature anomalies and obtain information on the surrounding obstacles. This paper proposes a visual saliency detection method for hypertemperature in three-dimensional space through dual-source images. The key novelty of this method is that it can achieve accurate salient object detection without relying on high-performance hardware equipment. First, the redundant point clouds are removed through adaptive sampling to reduce the computational memory. Second, the original images are merged with infrared images and the dense point clouds are surface-mapped to visually display the temperature of the reconstructed surface and use infrared imaging characteristics to detect the plane coordinates of temperature anomalies. Finally, transformation mapping is coordinated according to the pose relationship to obtain the spatial position. Experimental results show that this method not only displays the temperature of the device directly but also accurately obtains the spatial coordinates of the heat source without relying on a high-performance computing platform.

## 1. Introduction

In the path planning of mobile robots, it is common to construct a map using the dynamic vision fusion of cameras and multi-sensors [1,2,3]. In a specific industrial environment, the robot needs to monitor the temperature of the equipment and work in the area of abnormal temperature points. The existing neural network control method shows high stability [4,5,6], but it also needs to accurately find the location of the abnormal temperature’s point. Traditionally, using a visible-light binocular camera to reconstruct the target is not possible, because it cannot accurately operate on the abnormal temperature point area [7,8,9,10]. At present, the most commonly used temperature detection methods use sensor contact measurements [11,12,13]. However, there are installation and use problems in engineering applications, so non-contact space measurements can be used to solve the installation problem. Visual target detection can solve this problem.

In the field of target detection, deep learning is a commonly used technology. At present, in 2D target detection, many methods of optimizing the structure of deep convolutional neural networks improve the accuracy of target detection [14,15,16], such as fully convolutional networks (FCN), progressive fusion [17], multi-scale depth encoding [18], and data set balancing and smearing methods [19,20]. In mobile robot navigation, precise positioning of the target often requires obtaining spatial coordinates. The depth camera can be used to obtain depth information for 2.5D target positioning. The deep network also plays an important role in this field. The variational autoencoder [21,22], the adaptive window and weight matching algorithm [23], the deep purifier, and the feature learning unit greatly improve the accuracy of detection. However, deep learning requires more sophisticated hardware and relies on a large number of training samples [24,25,26,27].

With the development of 3D reconstruction technology, the application of 3D reconstruction technology in real life has become extensive, attracting the attention of many experts and scholars [28,29]. Commonly used 3D visual reconstruction methods include feature extraction and matching, sparse point cloud reconstruction, camera pose solution, dense point cloud reconstruction, and surface reconstruction [30,31,32,33]. Through the research of different experts and scholars, related technologies such as feature matching, depth calculation, and mesh texture reconstruction have made great breakthroughs, which have resulted in a higher degree of reduction in visual 3D reconstruction [34,35,36]. 

The method proposed in this paper mainly uses ordinary and infrared cameras to take pictures of targets and then sparse point cloud reconstruction through ordinary pictures to obtain the pose of the camera when imaging. Then, image fusion is performed on ordinary pictures and infrared pictures. The original camera’s internal and external parameters do not change. The original image can be replaced with the fusion image to surface-map the dense point cloud in order to generate a three-dimensional surface. In addition, a three-dimensional reconstruction target that visually displays the surface temperature is obtained [37,38,39]. This paper uses an adaptive random sampling algorithm to obtain the main texture features, remove redundant point clouds, and finally, use the depth confidence to filter the wrong point clouds [40,41,42].

**Figure 1:**
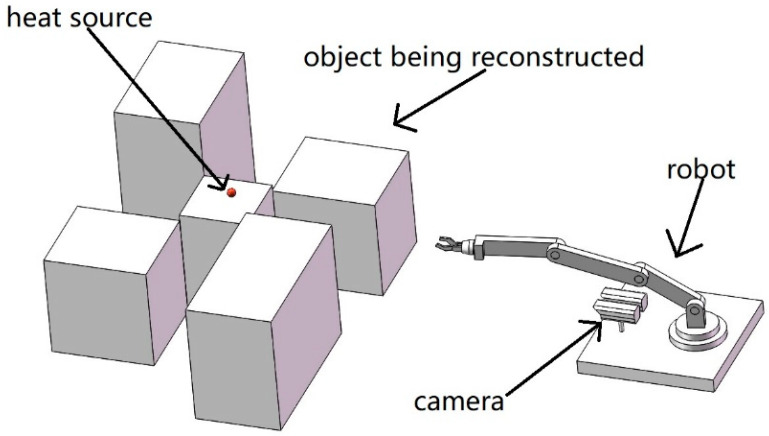
Robot operation diagram.

To reduce the calculation cost and dependence on training samples, this paper mainly uses the characteristics of infrared images to detect the center coordinates of the heat source. First, the infrared images are pre-processed by channel extraction and image segmentation. Then, the position of the two-dimensional plane temperature abnormal points is detected. Finally, the coordinate transformation is calculated based on the camera’s imaging pose relationship in order to calculate its spatial coordinates [43,44,45]. Therefore, it is possible to use the reconstructed target as an obstacle to plan the movement path of the robot and to work on the temperature abnormal point area according to the obtained spatial coordinate information. The schematic diagram is shown in Figure 1. The robot rotates around the target center once to reconstruct a complete target and quickly finds the center position of the heat source that needs to be operated using the above method.

## 2. Materials and Methods

The process of sparse point cloud reconstruction is as follows: Feature extraction, feature matching, elimination of mismatched pairs, 3D point cloud initialization, and camera pose calculation. Among these steps, the image mismatch elimination and pose solution have a great impact on the sparse point cloud reconstruction effect. The text uses the random sample consensus (RANSAC) algorithm to remove false matches and the beam adjustment method to recalculate the camera pose. The visible light camera used in this article is a 200W pixel POE DS-2CD3T25-I3 with a focal length of four millimeters. The device was manufactured by HIKVISION Company in Hangzhou, China.

### 2.1. Reconstruction of the Sparse Point Cloud to Obtain the Camera Attitude

#### 2.1.1. Use of the Scale-Invariant Feature Transform (SIFT) Algorithm to Find Feature Points

The process of sparse point cloud reconstruction includes feature extraction, feature matching, elimination of mismatched pairs, 3D point cloud initialization, and camera pose calculation. Among these steps, the image mismatch elimination and pose solution have the greatest impact on the sparse point cloud reconstruction effect. The text uses the RANSAC algorithm to remove false matches and the beam adjustment method to recalculate the camera pose.

To realize 3D reconstruction, the feature points of the picture first need to be extracted. The scale-invariant feature transform (SIFT) algorithm is a computer vision algorithm that is used to detect and describe local features of images, find extreme points in the interscale, and extract their position, scale, and rotation invariants. It is divided into the following four steps:

- Multi-scale spatial extreme point detection: This searches image locations on all scales and uses Gaussian differential functions to identify potential rotation invariants and scale candidate points.
- Accurate positioning of key points: After determining candidate positions, a high-precision model is fitted to determine the scale and position. The stability of key points is used as the basis for selection.
- Calculation of the main direction of key points: Based on the local gradient direction of the image, each key point obtains one or more directions. In the future, the image processing will be transformed relative to the key-point scale, direction, and position to ensure the invariance of the transformation.
- Descriptor construction: In the field of key points, the direction of local gradients is measured according to the scale selected above, and these gradients are transformed into another representation.

**Figure 2:**
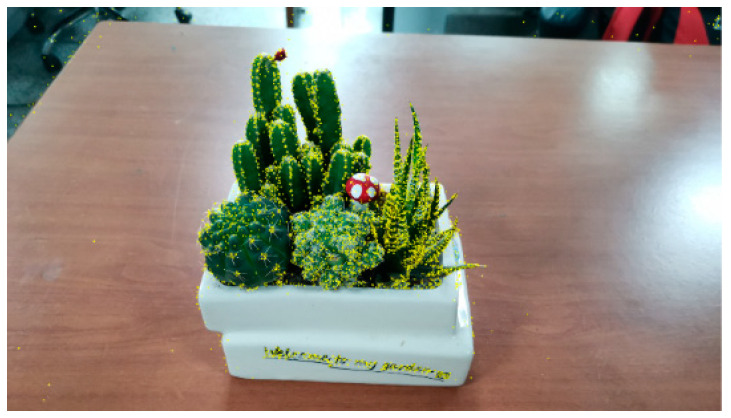
Scale-invariant feature transform (SIFT) feature point extraction results.

The effect of feature point extraction is shown in Figure 2.

This shows the reconstruction of a potted plant on a 3.0 GHz CPU desktop computer, selecting 30 consecutive shots at a resolution size of 4000×3000 ppi. The maximum calculation memory required during the reconstruction process, before using the adaptive sampling algorithm, is 5.3 GB. After adapting to the sampling algorithm, it is 3.2 GB, which proves that the algorithm effectively reduces the memory required for calculation.

#### 2.1.2. Error Matching Elimination Based on the RANSAC Algorithm

There will be matching errors after feature matching. RANSAC is a commonly used error elimination algorithm. The grid-based motion (GMS) [46] algorithm, recently proposed by scholars, can match features in a short time and is very robust. It can remove wrong matches to a certain extent. However, the original author notes that the GMS algorithm is suitable for supplementing the RANSAC algorithm but not replacing it. Therefore, this article mainly uses the RANSAC algorithm to eliminate wrong feature matching. The algorithm works by using Equation (2) as the cost function to iteratively update the sample set.

(1) $$s\left[\begin{array}{c} x^{\prime }\\ y^{\prime }\\ 1 \end{array}\right]=\left[\begin{array}{ccc} h_{11} & h_{12} & h_{13}\\ h_{21} & h_{22} & h_{23}\\ h_{31} & h_{32} & h_{33} \end{array}\right]\left[\begin{array}{c} x\\ y\\ 1 \end{array}\right]$$

(2) $$\overset{n}{\underset{i=1}{\sum }}{\left(x_{i}{}^{\prime }\frac{h_{11}x_{i}+h_{12}y_{i}+h_{13}}{h_{31}x_{i}+h_{32}y_{i}+h_{33}}\right)}^{2}+{\left(y_{i}{}^{\prime }\frac{h_{21}x_{i}+h_{22}y_{i}+h_{23}}{h_{31}x_{i}+h_{32}y_{i}+h_{33}}\right)}^{2}$$

In the above formula,  (x,y) represents the corner position of the target image, $\left(x^{\prime },y^{\prime }\right)$ is the corner position of the scene image, s is the scale parameter, and H is a 3 × 3 homography matrix.

**Figure 3:**
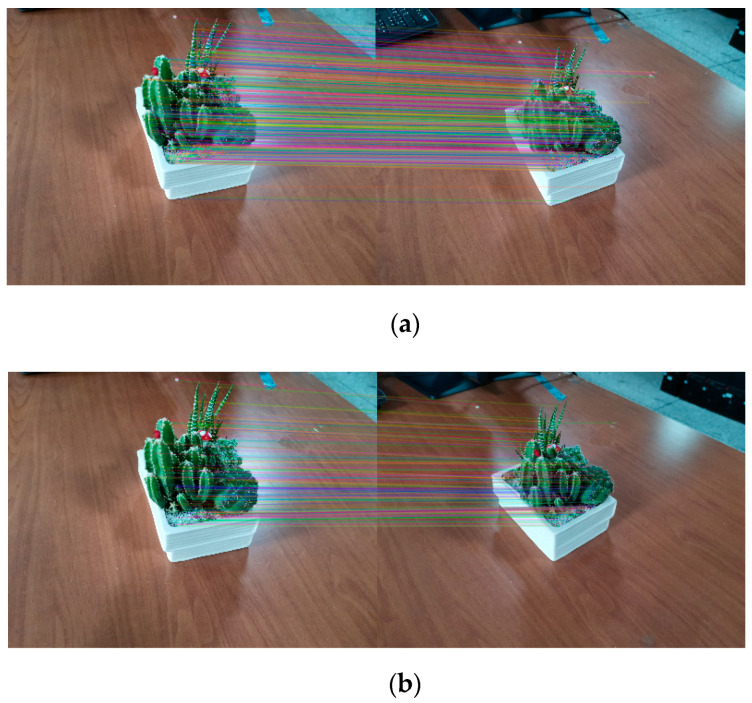
Comparison of algorithm effects, where (**a**) is the original matching effect diagram and (**b**) is the error matching elimination diagram of the random sample consensus (RANSAC) algorithm.

Error matching elimination based on RANSAC is shown in Figure 3.

#### 2.1.3. The Position Pose of the Phase Machine Is Solved by the Beam Adjustment Method

**Figure 4:**
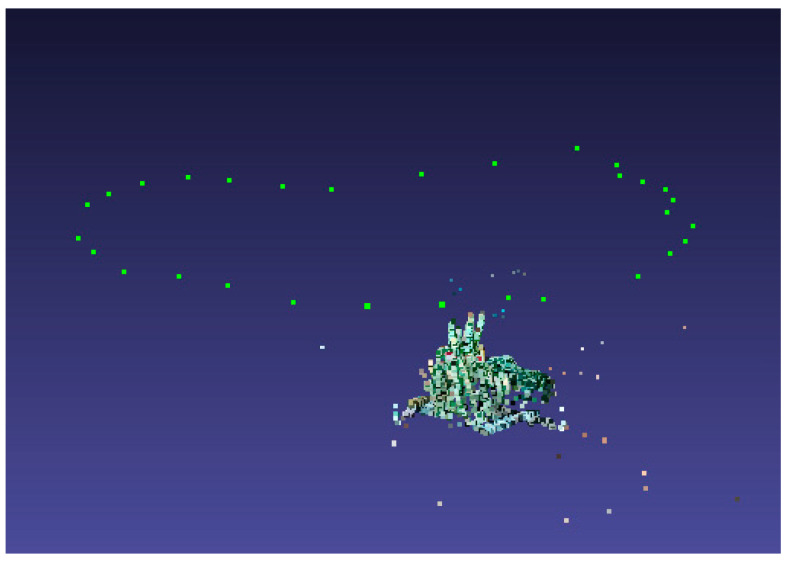
Schematic diagram after the camera pose calculation.

After the image alignment, the 3D point cloud and camera pose can be obtained. However, there will be interference noise when calculating the position and the 3D point, and there will be significant error in the subsequent calculation. Therefore, bundle adjustment is used to reduce the error [9], and the P matrix and F matrix of each picture after correction can be obtained. The reprojection error is defined as:

(3) $$E=\underset{j}{\sum }\rho_{j}\left(\pi \left(P_{C},X_{k}\right)-x_{j}{}^{2}\right)$$

where π is a projection matrix from three-dimensional to two-dimensional, $\rho_{j}$ is a kernel function, and $\pi \left(P_{C},X_{k}\right)-x_{j}{}^{2}$ is a cost function. Figure 4 shows the sparse point cloud obtained after the bundle adjustment (BA) algorithm is used to solve the position pose. The green dot is the posture of the solved camera.

### 2.2. Three-Dimensional Surface Generation

#### 2.2.1. Adaptive Random Sampling

- A pixel point $\hat{x_{i}}$ is randomly selected from the obtained point cloud image. $D_{i}\left(x_{i}\right)$ is the depth value of the pixel point and is inversely mapped into the three-dimensional space according to Equation (4). The tangent plane $P\left(\hat{x_{i}}\right)$ is obtained according to the normal direction. $K_{i}$ is the camera internal parameter, $R_{i}$ is the rotation matrix, and $T_{i}$ is the translation vector.
  (4) $$P\left(\hat{x_{i}}\right)=R_{i}{}^{T}\left(K^{-1}D_{i}\left(\hat{x_{i}}\right){\left[\hat{x_{i}}1\right]}^{T}-T_{i}\right)$$

Specific steps are as follows:

- Expand outwards with $\hat{x_{i}}$ as the center, expand the radius r one pixel at a time, and calculate the three-dimensional coordinates $P\left(x_{i}{}^{\prime }\right)$ of each pixel $x_{i}{}^{\prime }$ in the expansion range.
- Calculate the distance $d_{i}$ of each pixel $\;x_{i}{}^{\prime }$ to the tangent plane within the current expansion range, and set the threshold size as $t_{d}$. If $d_{i}\leq t_{d}$, the pixel point can be considered to be in the smooth area, and the point can be removed.
- When the expansion radius *r* is larger than the maximum expansion radius $r_{max}$, or a point cloud of a certain proportion of $p_{i}$ in the expansion range is removed, the expansion stops. $r_{max}$ and $p_{i}$ are tunable parameters. They can be determined according to the point cloud redundancy. During debugging, it is found that there are still many redundant point clouds after culling. $r_{max}$ can be increased and $p_{i}$ can be decreased. If the point cloud is over-eliminated, the parameter adjustment method is reversed.
- Then, randomly select a pixel point and repeat the above steps until all the sampling points in the current 3D point cloud image are sampled.

#### 2.2.2. Deep Confidence Removes the Cloud of Error Points

(5) $$E_{d}\left(P\left(\hat{x_{i}}\right)\right)=\frac{\sum_{t^{\prime }\epsilon N\left(t\right)}{\left|\left|D_{i}\left(\hat{x_{i}}\right)-D_{i}\left({\hat{x}}_{i\to i^{\prime }}\right)\right|\right|}^{2}}{\left|N\left(i\right)\right|}$$

**Figure 5:**
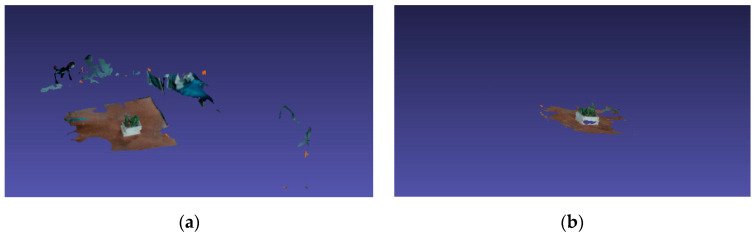
Effect before and after filtering, where (**a**) is the picture before removing the redundant point cloud, and (**b**) is the picture after removing the redundant point cloud.

The above formula is the depth value estimation of the point cloud, i.e., the larger the estimated value, the smaller the error value and the higher the reliability. Among these values, $\;E_{d}\left(P\left(\hat{x_{i}}\right)\right)$ is the depth value estimation of two adjacent frames, ${\hat{x}}_{i\to i^{\prime }}$ represents the projection point of the $i^{\prime }$ pixel projected by the current pixel, and  N(i) represents the number of frames taken. The specific steps are as follows:

- The point cloud for the current frame *k* is sorted from high to low according to the estimated value, and the confidence threshold $\varepsilon_{d}$ is set, starting from the point where the estimated value is the smallest. If $E_{d}\left(P\left(\hat{x_{i}}\right)\right)<\varepsilon_{d}$, the point is eliminated, the calculation continues until $E_{d}\left(P\left(\hat{x_{i}}\right)\right)>\varepsilon_{d}$ stops, and the remaining point clouds are stored in the sequence $S_{k}$. Then, the same calculation is performed on the next frame point cloud image until the point cloud image is calculated and the sequence set $S=\{S_{k}|k=1,\cdots ,n\}$ is obtained.

- Starting from the k frame depth map, all three-dimensional points $\hat{x_{i}}$ are mapped to ${\hat{x}}_{i+1}$ on the *k* + 1 frame. Compare the estimated values of the two points, the s.

- maller three-dimensional coordinates of the larger estimated points of the estimated values, and so on, until all depth maps are completed.
- The three-dimensional sampling points of all depth maps are intersected to obtain the final three-dimensional point cloud image. Then, perform the mesh reconstruction and mesh texture generation on the filtered dense point cloud. The effect before and after filtering is shown in Figure 5.

**Figure 6:**
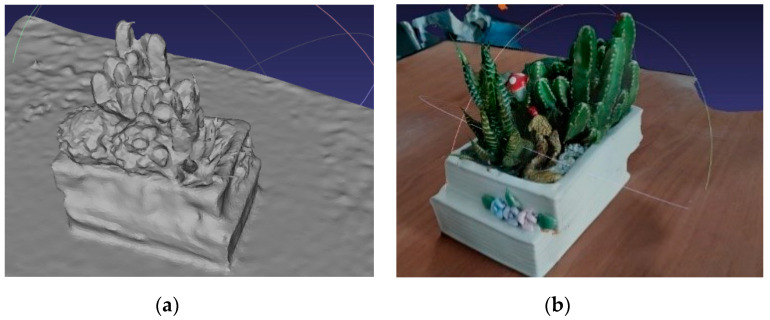
Surface reconstruction details, where (**a**) is the picture before the sticker and (**b**) is the picture after the sticker.

The reconstruction details are shown in Figure 6.

### 2.3. Image Fusion

After reconstructing the sparse point cloud, the camera parameters are obtained. The original image can be corrected for distortion. The infrared image can be calibrated and corrected by itself. The image registration error is shown in the following formula:

(6) $$\sigma_{x}=\frac{f\cdot d_{x}}{l_{pix}}\left(\frac{1}{D_{target}}-\frac{1}{D_{optimal}}\right)$$

where f is the focal length, $l_{pix}$ is the pixel size, and $d_{x}$ is the baseline length.$\;D_{optimal}$ is the target distance, and the alignment error of the image is zero. Only objects that are far away from the camera will be precisely aligned.

#### 2.3.1. Calculate Scale Factor

As the focal length and resolution of infrared and visible images are different, the imaging size of objects in space from the two camera types is not consistent. At the same time, the optical center of the hardware systems of the two camera types deviates in the Y direction. Therefore, it is not easy to scale the image by focal length.

The method adopted in this paper calculates the pixel difference between two corner points in infrared and visible images by using the checkerboard calibration board to obtain the image scale.

(7) $$scale=\frac{infrared_{n}-infrared_{n-1}}{visible_{n}-visible_{n-1}}$$

It is assumed that the checkerboard calibration board corner with k line, l column, namely kl, is accumulated. n is the corner number on the checkerboard, the upper left corner is minimum 1, and the lower right corner is the maximum kl. The values increase from left to right, and from top to bottom,$\;infrared_{n}$ is the x or y coordinate of the corner n on the infrared image, and $visible_{n}$ is the X or Y coordinate of the corner *n* on the visible light image.

#### 2.3.2. Relative Offset of the Image

**Figure 7:**
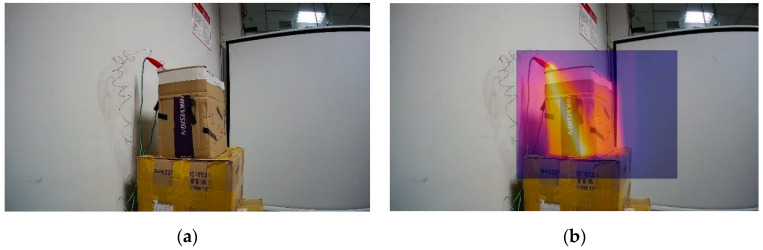
2D fusion picture, where picture (**a**) is the picture before fusion and picture (**b**) is the picture after fusion.

The factor scale is used to realize the unification of space objects in infrared and visible images. Then, the same corner point on the checkerboard is selected to calculate the relative offset of infrared and visible images.

(8) $$X_{diff}={infrared}_{x}-{visible}_{x}$$

(9) $$Y_{diff}=infrared_{y}-visible_{y}$$

where $\;X_{diff}$ and $Y_{diff}$ are the offsets required for each pixel in the infrared image. The RGB color model is a color standard in the industrial world. It obtains various colors by changing the three-color channels of red (R), green (G), and blue (B) and by superimposing each on others. After the completion of each pixel offset, the values of the three channels of RGB of the infrared and visible pixel pairs in the same coordinate can be fused, and the fusion effect is shown in Figure 7. Figure 7 is the heating plate placed in the carton. An infrared camera with a resolution of 384×288 ppi is used. The infrared camera and visible light camera take pictures at the same time.

**Figure 8:**
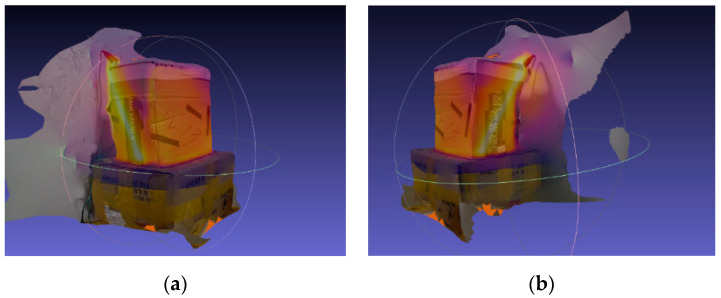
Schematic representation of temperature surface reconstruction, where (**a**) is reconstructed position 1 and (**b**) is reconstructed position 2.

The camera pose is calculated based on the reconstructed sparse point cloud, and all the fused pictures are surface-reconstructed. The 3D reconstruction effect of the temperature display is shown in Figure 8.

### 2.4. 3D Target Detection

**Figure 9:**
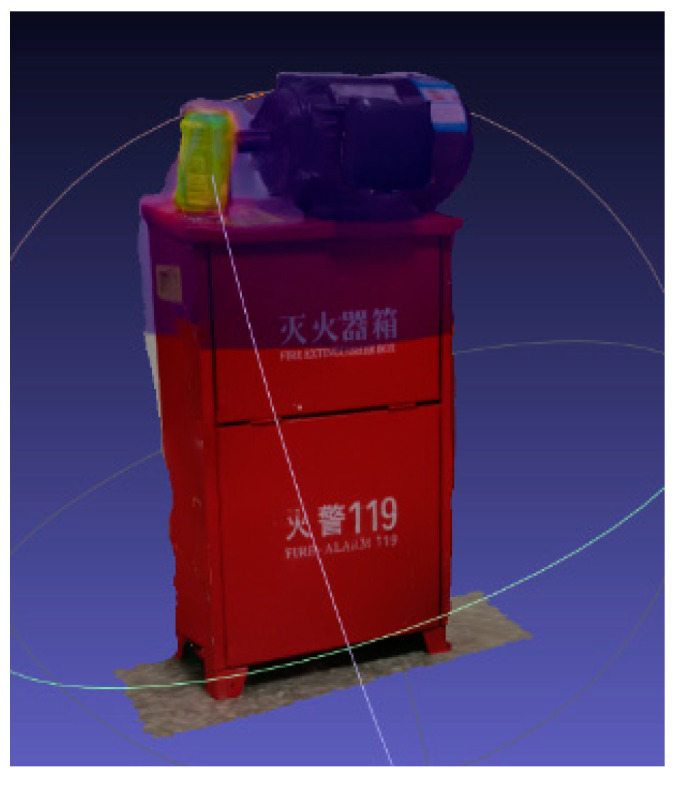
Detection target.

As shown in Figure 9, in this experiment, a high-temperature bottle is used as the temperature abnormal region of the overall device, and its spatial coordinates need to be calculated.

#### 2.4.1. Target Detection of the Heat Source

In the infrared picture, the pixel temperature generated by the detection is proportional to the R channel value, so the image can be preprocessed first. The R channel value size of the original image is extracted, and all pixels are sorted according to the R value. However, noise in the image is unavoidable and will interfere with the sorting results. To avoid incorrect sorting, the extracted image can be cut and divided into sub-regions. The size of the region can be determined according to the input original image size. Then, the average value of the R channel in each area is calculated, and the area is sorted according to the average value to obtain the R channel size set of each area $R_{agg}=\left\{R_{1},R_{2},R_{3},\cdots R_{n}\right\}$, assuming $R_{max}$ is its maximum value.

After the infrared image preprocessing is complete, the R channel value of each small area can be obtained. To allow the detection frame to be adaptively scaled, the size of the heat source needs to be calculated, so small squares (that meet the conditions) can be calculated and recorded for each small area location. The criteria are:

(10) $$R_{i}>k*R_{max}$$

(11) $$size_{r}=size_{p}*\;p_{r}$$

**Figure 10:**
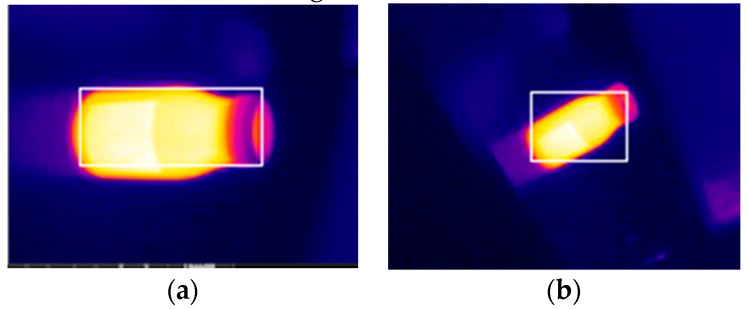
Heat source detection, where (**a**) is position 1 and (**b**) is position 2.

Among them, $R_{i}$ represents the value of the R channel region, and k is a proportionality coefficient that needs to be adjusted according to specific conditions. After calculating the situation of each sub-region, each region can be assessed, in order from left to right and top to bottom. Each sub-region is set to be square. The size of each sub-region $size_{r}$ can be determined according to Equation (11), where $size_{p}$ is the size of the infrared image used for detection, the proportion of $p_{r}$ sub-regions, and $p_{r}$ is an adjustable parameter. If four of the eight regions around the area meet the conditions, that area is a sub-area within the heat source range, and the position coordinate is recorded and evaluated. Finally, the size of the heat source border can be obtained from the coordinate position. The effect is shown in Figure 10.

#### 2.4.2. Coordinate Transformation Mapping in 3D Space

**Figure 11:**
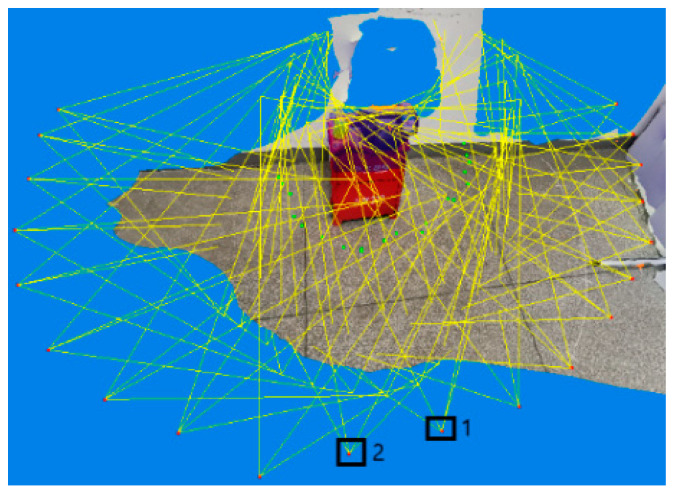
Camera imaging pose.

**Figure 12:**
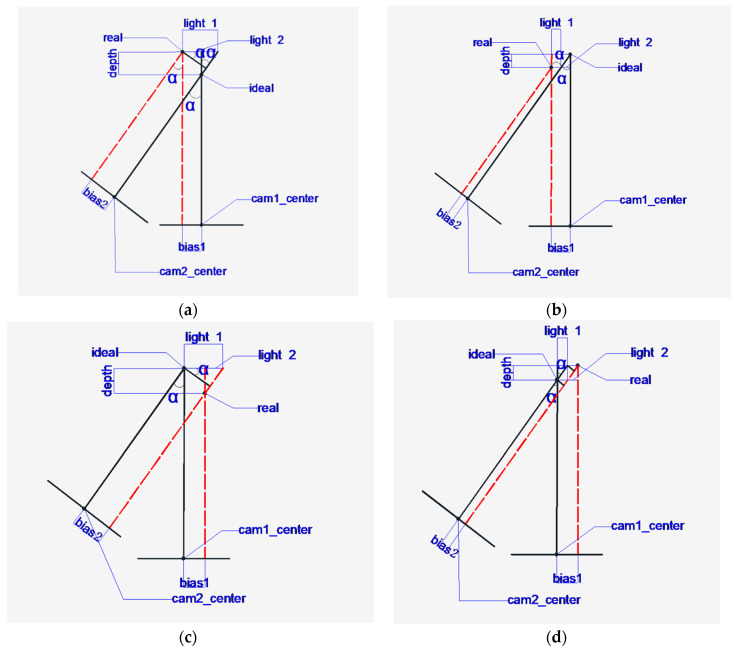

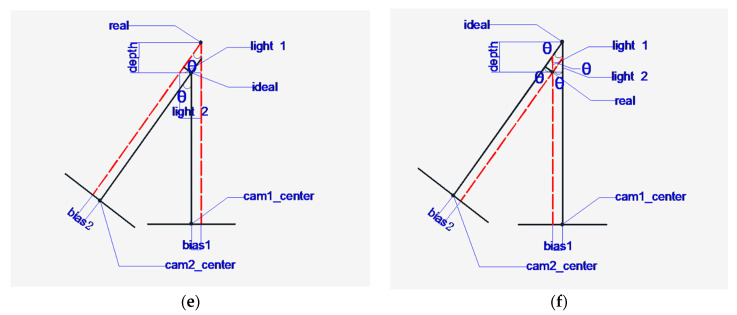
Schematic diagram of the ideal position and the actual position, where (**a**–**f**) corresponds to the situation of six actual heat sources relative to the ideal heat source.

After the detection of the heat source target, the coordinates of the heat source center in each infrared picture can be obtained; because the shoot is a head-up relationship, the horizontal deviation and the height deviation can also be obtained. The steps are as follows: Take the center of the first picture as the center point of the space and choose another angle during the shoot as the second position. As shown by two positions in Figure 11, calculate the deviation between the actual heat source and the ideal heat source. The following situations can occur:

Figure 12 is a top view of various situations. Taking Figure 12a as an example, cam1_center and cam2_center are the imaging center points of the camera at two positions, “ideal” is the most central position of the heat source processing experiment and is the intersection of the two imaging centers, and “real” is the actual position of the heat source. When the heat source reaches the imaging plane, the distance from the center of the camera is $bias_{1}\;$ and $bias_{2}$, where α is the angle of rotation of the second position relative to the first position. According to its geometric relationship, the rest of the same angle, that is, the angle shown in the figure, is obtained according to the geometric relationship.

(12) $$x=bias_{1}\;z=\left(z_{1}+z_{2}\right)/2\;light_{1}=bias_{2}/cos\alpha \;light_{2}=light_{1}-bias_{1}\;y=depth\;depth=light_{2}/tan\alpha$$

In the above formula, *z* is the height position of the heat source, and $z_{1}$ and $z_{2}$ are the deviations from the origin of the space coordinates at the heights taken at the two positions. In order to reduce the operation error, the average of the two positions is taken as the height deviation. $light_{1}$ and $light_{2}$ are the distances in the calculation of geometric relations, respectively. According to the above formula, the head-up deviation *x*, depth deviation *y*, and height deviation *z* can be obtained. As the coordinates in the actual space of the idea are already known, the space coordinates of the actual heat source can be calculated.

**Figure 13:**
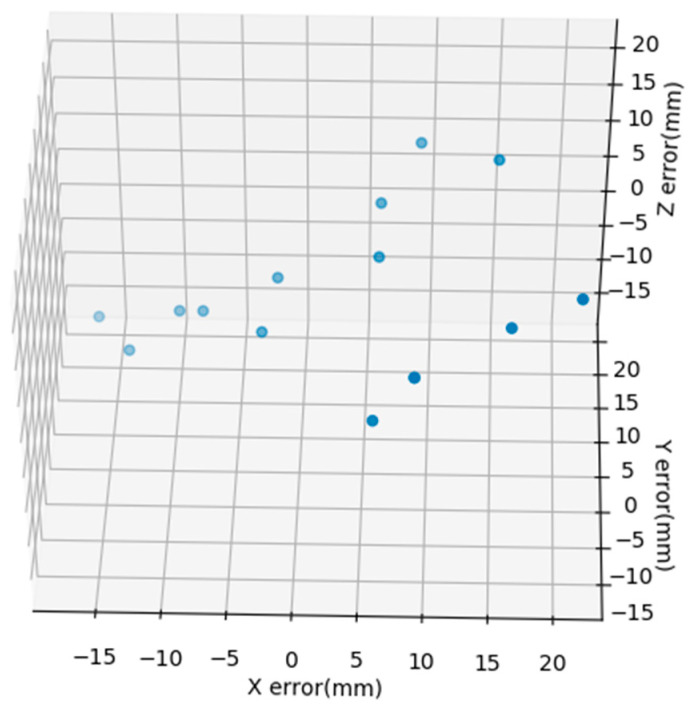
Camera imaging pose.

Although detection speed has been greatly improved by the enhanced convolutional neural network structure, it still cannot provide high-precision results, and relies on high-performance GPUs. The method in this paper conducted 15 experiments, only running on a 3.0 GHz desktop computer, using the thermos randomly placed in the above figure as a simulated heat source. The camera is 10 m away from the ideal heat source. The error values were obtained from the actual measured coordinates and calculated coordinates. The error results of the experiment are shown in Figure 13. It can be seen from the experimental results that the error value is within ±20 mm, with high accuracy, and the calculation speed is 20 ms, which meets the detection requirements of industrial equipment. 

## 3. Conclusions

The experimental results demonstrate that the method proposed in this paper can fuse target surface temperature information captured by infrared cameras into a three-dimensional point cloud while ensuring the accuracy and speed of the reconstruction and that the reconstructed object can intuitively display its surface temperature. The spatial coordinates of the heat source are calculated using the spatial transformation mapping relationship of the infrared picture. The experimental results demonstrate that the algorithm is highly accurate and meets the requirements of robot navigation and positioning.

## 4. Patents

A 3D reconstruction method based on point cloud optimization sampling; a 3D surface temperature display method based on infrared and visible image fusion is presented; the invention relates to a method for detecting the heat source center in three-dimensional space.

## References

[1] Fan Y., Lv X., Lin J., Ma J., Zhang G., Zhang L., Correction: Zhang G. (2019). Autonomous Operation Method of Multi-DOF Robotic Arm Based on Binocular Vision. Appl. Sci..

[2] Kassir M.M., Palhang M., Ahmadzadeh M.R. (2018). Qualitative vision-based navigation based on sloped funnel lane concept. Intell. Serv. Robot..

[3] Li C., Yu L., Fei S. (2020). Large-Scale, Real-Time 3D Scene Reconstruction Using Visual and IMU Sensors. IEEE Sens. J..

[4] Yang C., Jiang Y., He W., Na J., Li Z., Xu B. (2018). Adaptive Parameter Estimation and Control Design for Robot Manipulators with Finite-Time Convergence. IEEE Trans. Ind. Electron..

[5] Yang C., Peng G., Cheng L., Na J., Li Z. (2019). Force Sensorless Admittance Control for Teleoperation of Uncertain Robot Manipulator Using Neural Networks. IEEE Trans. Syst. ManCybern. Syst..

[6] Peng G., Yang C., He W., Chen C.P. (2020). Force Sensorless Admittance Control with Neural Learning for Robots with Actuator Saturation. IEEE Trans. Ind. Electron..

[7] Mao C., Li S., Chen Z., Zhang X., Li C. (2020). Robust kinematic calibration for improving collaboration accuracy of dual-arm manipulators with experimental validation. Measurement.

[8] Xu L., Feng C., Kamat V.R., Menassa C.C. (2020). A scene-adaptive descriptor for visual SLAM-based locating applications in built environments. Autom. Constr..

[9] Yang C., Wu H., Li Z., He W., Wang N., Su C.Y. (2018). Mind Control of a Robotic Arm with Visual Fusion Technology. IEEE Trans. Ind. Inform..

[10] Lin H., Zhang T., Chen Z., Song H., Yang C. (2019). Adaptive Fuzzy Gaussian Mixture Models for Shape Approximation in Robot Grasping. Int. J. Fuzzy Syst..

[11] Shen S. (2013). Accurate Multiple View 3D Reconstruction Using Patch-Based Stereo for Large-Scale Scenes. IEEE Trans. Image Process..

[12] Wang L., Li R., Sun J., Liu X., Zhao L., Seah H.S., Quah C.K., Tandianus B. (2019). Multi-View Fusion-Based 3D Object Detection for Robot Indoor Scene Perception. Sensors.

[13] Yamazaki T., Sugimura D., Hamamoto T. Discovering Correspondence Among Image Sets with Projection View Preservation For 3D Object Detection in Point Clouds. Proceedings of the 2018 IEEE International Conference on Acoustics, Speech and Signal Processing (ICASSP).

[14] Zhou Y., Tuzel O. VoxelNet: End-to-End Learning for Point Cloud Based 3D Object Detection. Proceedings of the IEEE Conference on Computer Vision and Pattern Recognition.

[15] Fu K., Zhao Q., Gu I.Y., Yang J. (2019). Deepside: A general deep framework for salient object detection. Neurocomputing.

[16] Wang W., Shen J. (2018). Deep Visual Attention Prediction. IEEE Trans. Image Process..

[17] Tang Y., Zou W., Hua Y., Jin Z., Li X. (2020). Video salient object detection via spatiotemporal attention neural networks. Neurocomputing.

[18] Zhao J.X., Liu J.J., Fan D.P., Cao Y., Yang J., Cheng M.M. EGNet:Edge Guidance Network for Salient Object Detection. Proceedings of the IEEE International Conference on Computer Vision.

[19] Ren Q., Hu R. (2018). Multi-scale deep encoder-decoder network for salient object detection. Neurocomputing.

[20] Fan D.P., Cheng M.M., Liu J.J., Gao S.H., Hou Q., Borji A. Salient Objects in Clutter: Bringing Salient Object Detection to the Foreground. Proceedings of the European Conference on Computer Vision (ECCV).

[21] Zhang J., Yu X., Li A., Song P., Liu B., Dai Y. Weakly-Supervised Salient Object Detection via Scribble Annotations. Proceedings of the IEEE/CVF Conference on Computer Vision and Pattern Recognition.

[22] Zhang J., Fan D.P., Dai Y., Anwar S., Saleh F.S., Zhang T., Barnes N. UC-Net: Uncertainty Inspired RGB-D Saliency Detection via Conditional Variational Autoencoders. Proceedings of the IEEE/CVF Conference on Computer Vision and Pattern Recognition.

[23] Wang Z., Liu J. (2020). Research on flame location based on adaptive window and weight stereo matching algorithm. Multimed. Tools Appl..

[24] Fan D.P., Ji G.P., Sun G., Cheng M.M., Shen J., Shao L. (2019). Rethinking RGB-D Salient Object Detection: Models, Datasets, and Large-Scale Benchmarks. IEEE Trans. Neural Netw. Learn. Syst..

[25] Hu Q.H., Huang Q.X., Mao Y., Liu X.L., Tan F.R., Wang Y.Y., Yin Q., Wu X.M., Wang H.Q. (2019). A near-infrared large Stokes shift probe based enhanced ICT strategy for F- detection in real samples and cell imaging. Tetrahedron.

[26] Song W.T., Hu Y., Kuang D.B., Gong C.L., Zhang W.Q., Huang S. (2019). Detection of ship targets based on CFAR-DCRF in single infrared remote sensing images. J. Infrared Millim. Waves.

[27] Zhao X., Wang W., Ni X., Chu X., Li Y.F., Lu C. (2019). Utilising near-infrared hyperspectral imaging to detect low-level peanut powder contamination of whole wheat flour. Biosyst. Eng..

[28] Hruda L., Dvořák J., Váša L. (2019). On evaluating consensus in RANSAC surface registration. Comput. Graph. Forum.

[29] Qu Y., Huang J., Zhang X. (2018). Rapid 3D Reconstruction for Image Sequence Acquired from UAV Camera. Sensors.

[30] Aldeeb N.H., Hellwich O. (2020). 3D Reconstruction Under Weak Illumination Using Visibility-Enhanced LDR Imagery. Adv. Comput. Vis..

[31] Xie Q.H., Zhang X.W., Cheng S.Y., Lv W.G. (2019). 3D Reconstruction Method of Image Based on Digital Microscope. Acta Microsc..

[32] Zhang J., Zhang S.X., Chen X.X., Jiang B., Wang L., Li Y.Y., Li H.A. (2019). A Novel Medical 3D Reconstruction Based on 3D Scale-Invariant Feature Transform Descriptor and Quaternion-Iterative Closest Point Algorithm. J. Med. Imaging Health Inf..

[33] Zhang K., Yan M., Huang T., Zheng J., Li Z. (2019). 3D reconstruction of complex spatial weld seam for autonomous welding by laser structured light scanning. J. Manuf. Process..

[34] Zhang X., Zhao P., Hu Q., Wang H., Ai M., Li J. (2019). A 3D Reconstruction Pipeline of Urban Drainage Pipes Based on MultiviewImage Matching Using Low-Cost Panoramic Video Cameras. Water.

[35] Zheng Y., Liu J., Liu Z., Wang T., Ahmad R. (2019). A primitive-based 3D reconstruction method for remanufacturing. Int. J. Adv. Manuf. Technol..

[36] Zhu C., Yu S., Liu C., Jiang P., Shao X., He X. (2019). Error estimation of 3D reconstruction in 3D digital image correlation. Meas. Sci. Technol..

[37] Kiyasu S., Hoshino H., Yano K., Fujimura S. (1995). Measurement of the 3-D shape of specular polyhedrons using an M-array coded light source. IEEE Trans. Instrum. Meas..

[38] Pollefeys M., Nistér D., Frahm J.M., Akbarzadeh A., Mordohai P., Clipp B., Engels C., Gallup D., Kim S.J., Merrell P. (2008). Detailed Real-Time Urban 3D Reconstruction from Video. Int. J. Comput. Vis..

[39] Furukawa Y., Ponce J. (2009). Carved Visual Hulls for Image-Based Modeling. Int. J. Comput. Vis..

[40] Zhan Y., Hong W., Sun W., Liu J. (2019). Flexible Multi-Positional Microsensors for Cryoablation Temperature Monitoring. IEEE Electron Device Lett..

[41] Zhou H., Zhou Y., Zhao C., Wang F., Liang Z. (2018). Feedback Design of Temperature Control Measures for Concrete Dams based on Real-Time Temperature Monitoring and Construction Process Simulation. KSCE J. Civ. Eng..

[42] Zrelli A. (2019). Simultaneous monitoring of temperature, pressure, and strain through Brillouin sensors and a hybrid BOTDA/FBG for disasters detection systems. IET Commun..

[43] Sun H., Meng Z.H., Du X.X., Ang M.H. A 3D Convolutional Neural Network towards Real-time Amodal 3D Object Detection. Proceedings of the 2018 IEEE/RSJ International Conference on Intelligent Robots and Systems (IROS).

[44] Shen X.L., Dou Y., Mills S., Eyers D.M., Feng H., Huang Z. (2018). Distributed sparse bundle adjustment algorithm based on three-dimensional point partition and asynchronous communication. Front. Inf. Technol. Electron. Eng..

[45] Snavely N., Seitz S., Szeliski R. (2006). Photo tourism: Exploring photo collections in 3D. ACM Trans. Graph. (TOG).

[46] Bian J.-W. (2020). GMS: Grid-Based Motion Statistics for Fast, Ultra-robust Feature Correspondence. Int. J. Comput. Vis..

